# The ART approach: clinical aspects reviewed

**DOI:** 10.1590/S1678-77572009000700016

**Published:** 2009

**Authors:** Gustavo Fabián MOLINA, Ricardo Juan CABRAL, Jo E. FRENCKEN

**Affiliations:** 1 DDS, PhD, Department of Dental Materials, The Dental Faculty, National University of Córdoba and Catholic University of Córdoba, Argentina.; 2 DDS, PhD, Department of Dental Materials, The Dental Faculty, National University of Córdoba, Argentina.; 3 DDS, MSc, PhD, Department of Global Oral Health, College of Dental Sciences, Radboud University Nijmegen Medical Centre, The Netherlands.

**Keywords:** Atraumatic Restorative Treatment (ART), Glass-ionomer cements, Minimal intervention dentistry, Sealants, Restorations

## Abstract

The success of ART as a caries management approach is supported by more than 20 years of scientific evidence. ART follows the contemporary concepts of modern cariology and restorative dentistry. It challenges treatment concepts such as step-wise excavation and the need for complete removal of affected dentine. The ART approach so far has mainly used high-viscosity glass-ionomer as the sealant and restorative material. Cariostatic and remineralization properties have been ascribed to this material which requires further research to establish its clinical relevance. The adhesion of high-viscosity glass-ionomer to enamel in pits and fissures is apparently strong, as its remnants, blocking the pits and fissures, have been considered a possible reason for the low prevalence of carious lesion development after the glass-ionomer has clinically disappeared from it. encapsulated high-viscosity glass-ionomers may lead to higher restoration survival results than those of the hand-mixed version and should, therefore, not be neglected when using ART. Similarly, the use of resin-modified glass-ionomer with ART should be researched. The effectiveness of ART when compared to conventional caries management approaches has been shown in numerous studies. Proper case selection is an important factor for long-lasting ART restoration survival. This is based on the caries risk situation of the individual, the size of the cavity opening, the strategic position of the cavitated tooth and the presence of adequate caries control measures. As the operator is one of the main causes for failure of ART restorations, attending a well-conducted ART training course is mandatory for successful implementation of ART.

## INTRODUCTION

The Atraumatic Restorative Treatment (ART), by definition, has features that characterize this approach and differentiate it from what we know as “conventional” operative dentistry for the management of carious lesions. Frencken and Holmgren^26^ (1999) defined ART as a “maximally preventive and minimally invasive approach to arrest further progression of dental caries. It involves the removal of soft, completely demineralised carious tooth tissues with hand instruments, followed by the restoration of the cavity with an adhesive dental material that simultaneously seals the remaining pits and fissures that remain at risk.” Risk assessment is also the driving force behind the use of the preventive aspect of ART. This is achieved through sealing pits and fissures prone to development of carious lesions.

The purpose of this paper is to analyze and discuss the components that define ART, using published study outcomes, to discuss the contribution of ART to the management of carious lesion development in general and to identify issues that require further research.

## ART SEALANTS: AN EFFECTIVE MEASURE TO PREVENT CARIOUS LESION DEVELOPMENT

Fissure sealants have been accepted as effective tools for preventing carious lesion development in (newly) erupted molars and premolars exposed to potential caries-risk factors. They appear to be more effective than the common fluoride varnishes but the evidence is not substantial and is dependent upon local circumstances^30^.

Retention of a sealant is usually considered the most important variable indicating its effectiveness. Those who disagree with this view have postulated that its carious lesion preventive effect is the real endpoint and that sealant retention is merely its surrogate^26^. These two variables do not necessarily correlate well, as is shown in the following example. A comparison between ART sealants using two types of glass-ionomer in a high caries-risk population was carried out in Brazil^54^. The study showed a high preventive effect (98.5%) for both type of sealants, whilst the retention rates of both types was lower than 50% after 1 year. Obviously, the level of caries risk in an individual and the level of professionalism of the practitioner have an important impact upon the relative contributions of both variables to the effectiveness of a sealant.

**FIGURE-1:**
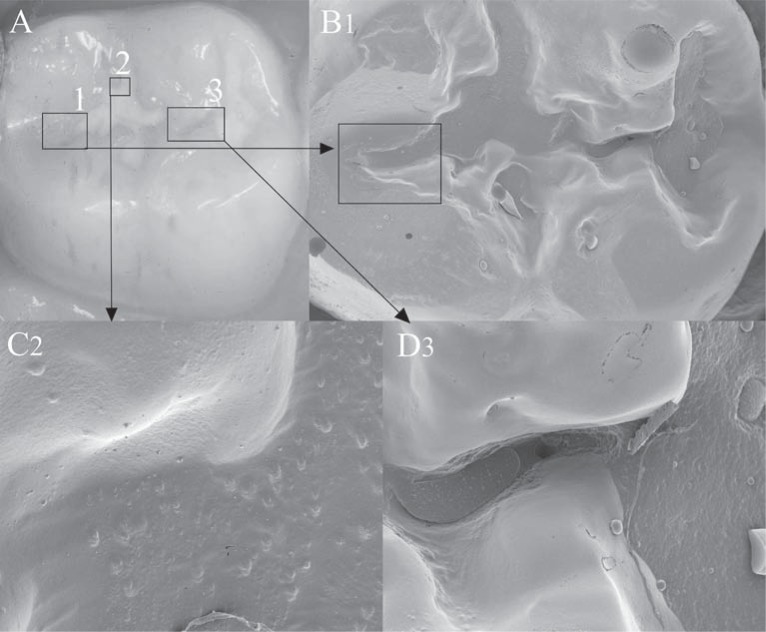
A) High-viscosity glass-ionomer (Fuji IX) sealant in tooth 47 after 12 years. The distal fissure appears to be clinically free of glass-ionomer material. B1) On the scanning electron microscopy (SEM) image (12x), glass-ionomer material is clearly visible till end of distal fissure. C2) Good adhesion of high-viscosity glass-ionomer to enamel (SEM: 100x). D3) Glass-ionomer material present in the fissure connecting the central with the mesial pit (SEM: 100x). The glass-ionomer sealant was clinically not visible in the fissure (Copyright: J. Frencken)

High-viscosity glass-ionomers are used in placing ART sealants. In the only comparative clinical trial published so far, they prevented carious lesion development in re-exposed pits and fissures of occlusal surfaces more effectively than resin composite sealants did^5^. Discussion continues as to whether such an effect can be ascribed to the fluoride release from the glass-ionomers used. However, some studies have shown that the fluoride release from glass-ionomers is low and clinically insignificant^55^. Others have demonstrated that glass-ionomer has a remineralising effect and ascribed this to its fluoride release^2^^,^^17^. Nevertheless, it appears that the view that their fluoride release is responsible for the preventive effect of glass-ionomer sealants may be based on insufficient evidence. A more plausible reason for its preventive effect over time could be related to the remnants of glass-ionomer left behind in the deeper parts of the pits and fissures, as was recently demonstrated by Frencken and Wolke^29^ (2010) (Figure 1). This feature had already been described by Mejare and Mjör^40^ (1990) and Williams, et al.^56^ (1996) as a possible explanation for the caries preventive effect in deep pits and fissures after the sealant material had clinically disappeared. Obviously, there is a need to further investigate and compare of glass-ionomer and other sealant materials regarding this characteristic. Results of the comparison would assist the dental practitioner to decide which sealant material to use in order to obtain a long-lasting caries preventive effect.

The meta-analysis by Van't Hof, et al.^53^ (2006) concluded that although the number of studies reporting on the retention and caries preventive effect of ART sealants was low, the retention of high-viscosity glass-ionomer ART sealants was higher than that of medium-viscosity glass-ionomer ART sealants. Furthermore, the caries preventive effect was high: 99%, 98% and 97% after 1, 2 and 3 years, respectively. This meta-analysis showed that only high-viscosity glass-ionomer should be used for sealing pits and fissures using ART.

## USING ART IN MANAGING CAVITATED DENTIN LESIONS

Hand instruments are used for cavity cleaning in accordance with ART. Although hand excavators have been used to clean cavities for more than a century, many dental practitioners resort solely to rotary equipment when "preparing and cleaning" a cavity, thinking that using hand instrumentation alone will lead to insufficient results. In light of this, issues related to the use of the ART approach will be discussed.

## HAND EXCAVATION VERSUS OTHER MEANS OF REMOVING CARIOUS TISSUES

Is the cavity clean enough after hand excavation to survive for long? A few *in-vitro* and *in-vivo* studies have provided some results. Bannerjee, et al.^3^ (2000) concluded, in an *in-vitro* multiple-caries removal measures comparison study, that using a chemomechanical caries removal gel, manipulated by hand instruments especially manufactured to ensure optimum cleaning of the tooth cavities, was the best way of removing carious tissues from an occlusal cavity. However, its disadvantage was the amount of time required to complete the procedure. This study concluded that the use of hand excavators was the most effective method of cleaning cavitated tooth cavities in permanent molars. A similar study, covering primary teeth, also showed hand excavators to be the most effective instruments for cleaning tooth cavities^14^. An *in-vivo* study demonstrated no difference in caries left behind in cavities treated with hand instruments and in those treated with a chemomechanical caries removal gel^42^.

Topaloglu-Ak, et al.^51^ (2009) compared survival rates of composite restorations performed in class II cavities in primary teeth, cleaned using hand instruments only (ART) and those cleaned with a chemomechanical caries removal gel. The restoration survival results were not significantly different from each other after 2 years. A pilot study, using the same two methods of cavity cleaning, after 12 months showed no significant differences in restoration survival results in permanent teeth restored with a high-viscosity glass-ionomer^4^.

On the basis of the available evidence it can be concluded that hand instruments, such as used with ART, are effective for cleaning cavitated dentine lesions. However, the size of the opening of the cavity appears to have an effect on the level of cleanliness of the cavity in occlusal surfaces^43^. The authors concluded that a cavity opening of at least Ø 1.6 mm was necessary for ensuring adequate removal of infected (decomposed) dental tissues.

## MICROORGANISMS LEFT IN THE CAVITY

A recently published critical review stated that cariogenic bacteria, once isolated from their source of nutrition by a restoration of sufficient integrity, either die or remain dormant and thus, pose no risk to the health of the tooth^50^. This implies that, in essence, there is no need to try to remove all microorganisms from within the cavity. If this is attempted, potentially remineralizable and sound dentine is sacrificed, which would inevitably lead to a reduction in the strength of the tooth. This argument is supported by Maltz, et al.^36^^,^^37^ (2002, 2007), who concluded that incomplete removal of carious affected (demineralised) dentin and subsequent restoration of the cavity with a material that seals the cavity tightly results in the arrest of the lesion. The authors suggested that complete removal of affected (demineralised) dentin is not essential for controlling the progression of dentine carious lesions.

Further support for the finding that microorganisms become inactive after the sealing of small dentine lesions is provided in a systematic review^45^. The review concluded that microorganisms left in small cavities declined in number over time. The authors suggested that sealing over small dentine lesion(s) in pits and fissures is an evidence-based treatment.

This evidence shows that when a cavity is securely restored with a material having a good and long-lasting bond to the cavity walls, micro-organisms unintentionally left behind will not restart the caries process. This does not, however, mean that cavities should be left full of infected (decomposed) dentine and then filled with a restorative material. The intention when using ART is to remove as much infected (decomposed) dentine from the cavity as possible, in order to create the largest possible intra-cavity surface for a secure bonding. Thus production of ART restorations follows the same principles as those of contemporary cariology and restorative dentistry^32^.

## STEPWISE-EXCAVATION VERSUS ONE-SESSION ART APPROACH

In managing deep carious lesions, the risk of pulp exposure during the removal of infected (decomposed) dentinal tissues led to development of a biological approach intended to preserve tooth tissues and promote the defence of the pulp by a total seal of the cavity and by the stimuli of calcium hydroxide cement. This approach is called “stepwise-excavation”^9^. This approach challenged the belief that the infected (decomposed) dentin had to be removed completely in order to eliminate any potential threat of infection. It demonstrated that it was possible to leave behind a bacterial component controlled by a dental material with healing properties^7^^,^^8^.

The stepwise excavation technique requires re-entering of the cavity to complete the removal of infected (decomposed) dentine, whereas ART uses only one step. The need for re-entering was investigated in an *in-vivo* study. At baseline and after 3 months, clinical, ultra-structural and chemical analysis was done of cavities in primary molars treated according to ART and filled with a glass-ionomer in one session. The results showed a large reduction in micro organisms, a more densely packed dentine structure and an increase in the calcium content. The authors concluded that a one-session approach creates favourable conditions for the healing process of affected (demineralised) dentine^38^. The application of the ART approach and its success over two decades raises the question as to whether stepwise-excavation is really needed.

Rickets, et al.^48^ (2006) conducted a systematic review to test the null hypothesis of no difference in the incidence of damage or disease of the pulp, progression of decay and longevity of restorations, irrespective of whether the removal of decay had been minimal (ultraconservative) or complete. The conclusion was that for reducing the risk of pulp exposure, partial caries removal is preferable to complete caries removal in the deep lesion. However, evidence related to the necessity of re-entering and excavating further was insufficient, although studies where this had not been done did not report adverse consequences. ART studies had not been included in this review. Knowing that particularly in deep carious lesions, infected (decomposed) dentine may be left behind during the ART procedure and considering the absence of reports of abscessed or extracted ART restorations, many ART studies do not support the need for removal of deep caries infected (decomposed) dentine and thus, for re-entry into the cavity.

## BOND STRENGTH OF RESTORATIVE MATERIALS USED WITH ART ON CARIES-AFFECTED DENTIN

From a pathological point of view, it appears that removal of all affected (demineralised) carious tissues from the cavity surfaces is unnecessary. However, to what extent does this situation affect the bonding of restorative materials to the cavity walls? How good is the bonding, of restorative materials used in the ART approach, to the treated dental tissues?

There is evidence which shows that the presence of caries-affected (demineralised) dentine may negatively affect the bonding of glass-ionomers to both enamel and dentin, regardless of the cavity preparation method^15^. The mean values regarding bond strength to caries-affected (demineralised) dentine may vary among different brands of glass-ionomer used. For example; it was reported that the mean bond strength to caries-affected (demineralised) dentine of three conventional glass-ionomers (one medium- and two high-viscosity) tested were lower than that of the resin-modified glass-ionomer used^46^.

If resin composite is chosen as the restorative material for ART, the presence of infected (decomposed) dentin may also influence the bond strength of the adhesive systems to dentine and enamel. Two studies comparing micro-tensile bond strength of different resin-based dentin adhesives over sound and caries-affected (demineralised) dentin concluded that values are higher when the remaining dental tissues are not affected by the caries process^12^^,^^22^. However, adhesion can be enhanced by means of rinsing solutions like sodium hypochlorite^49^ or 2% chlorhexidine digluconate^35^.

In conclusion, considering all the biological aspects discussed above, it is important to ensure that as much as possible of the infected, softened (decomposed) dental tissue is removed, in order to obtain adequate adhesion of the restorative material to the cavity walls over a long period, irrespective of the restorative material used.

## CASE SELECTION OF CAVITIES TREATABLE WITH ART

It is obvious that the cavity size, selection of restorative material, clinical skills and knowledge of the dental practitioner will determine the success of a restoration, whether conventional, ART or any other cavity cleaning method is used.

The meta-analysis showed that the highest survival rates for ART restorations using high-viscosity glass-ionomers were observed in single-surface cavities in both permanent and primary teeth, while high-viscosity glass-ionomer ART restoration survival rates of multiple-surface cavities in primary teeth needed further improvements^53^. Among the reasons given for clinical failure of ART restorations in multiple-surface cavities in primary teeth are those related to the restorative material used and the operator^28^. As an example of the latter serves a study that was carried out in a high-caries risk child population in the jungle of Surinam. Many (large) cavities were restored, using ART and a high-viscosity glass-ionomer. No reported preventive programme accompanied the restorative care. The survival of ART restorations after 3 years was low. About 34% of multiple-surface cavities were restored but blood and/or saliva had contaminated the cavity^52^. Under such adverse circumstances, good restorations, irrespective of the restorative approach and restorative material used, cannot be achieved. Other treatments like extraction, placing stainless steel crowns or cavity cleaning with a tooth brush and toothpaste would have perhaps been more appropriate^33^.

## RESTORATIVE MATERIALS USED WITH ART

According to the definition of restorative ART, the cavity should be filled with an adhesive material which seals the adjacent pits and fissures of the cavity in order to prevent carious lesion development. A number of features such as the sensitivity of the manipulation, the effectiveness of bonding to dental tissues, minimal dimensional changes after hardening and thermo-cycling (heating and cooling in wet conditions), fluoride release/uptake and the remineralisation potential, have to be analyzed to determine which restorative material is suitable for use with ART.

## RESIN COMPOSITES

Resin composites have not been used as a first choice for producing ART restorations and ART sealants, despite their good optical and mechanical properties. This is mainly because use of rotary equipment is required for an optimal performance of the material.

However, motivated by low survival rates of multiple-surface ART restorations in primary teeth, Ersin, et al.^23^ (2006) carried out a comparative study in class II ART- cleaned cavities, using a high-viscosity glass-ionomer and a resin composite self-etch dentin adhesive system (Xeno III). Although resin composite had higher survival rates, no statistically significant difference was observed between the two types of restoration after 2 years. Resin composite, in combination with the self-etch bonding liquid (Prompt L-Pop), was used to restore class II cavities in primary teeth cleaned according to ART and the results were compared with those of restorations prepared using rotary instrument. This study was carried out to investigate whether the use of resin composite would increase the survival rate of ART restorations using high-viscosity glass-ionomers in class II cavities in primary teeth^20^. After 2 years the survival of both types of restorations were distinctly lower than that reported for ART restorations in class II cavities using high-viscosity glass-ionomers reported in the meta-analysis^53^. In order to test whether the low survival of resin composite class II ART restorations in primary teeth was due to insufficient removal of infected (decomposed) dentine from these cavities, a trial was undertaken, in which ART was used for cleaning class II cavities in primary teeth, with and without the use of a chemomechanical caries removal gel, and restored with a resin composite and the self-etch bonding (Adper Prompt L-Pop)^51^. Results after 2 years showed distinctly lower survival percentages than that reported for ART restorations in class II cavities using high-viscosity glass-ionomers reported in the meta-analysis^53^.

The studies covering ART-cleaned class II cavities in primary teeth restored with a resin composite and a self-etch bonding have not led to a superior restoration survival percentage than that obtained for those restored with a high-viscosity glass-ionomer. Failure of the resin composite ART restorations was mainly attributed to the poor performance of the self-etch bondings used. This may not imply that high-viscosity glass-ionomer ART class II restorations in primary teeth are superior to comparable restorations with resin composite bonded with a 3-step system. However, it can be concluded that resin composite restorations can be produced with ART in class II cavities in primary teeth, and that the self-etch bonding systems used were of inferior quality.

## GLASS-IONOMER CEMENTS

Because of its biological, physical and chemical properties, the most suitable filling material according to ART definition is the glass ionomer cement. Particularly, its relatively slow setting time makes high-viscosity glass-ionomer the most appropriate material for use with ART. Several authors consider glass-ionomers to be “smart” restorative materials. A smart material is by definition a material possessing properties which may be altered in a controlled fashion by stimuli such as stress, temperature, moisture, pH, electricity or magnetic fields^39^.

Cariostatic and remineralising properties, identified in *in-vitro* studies, have frequently been ascribed to glass-ionomers but their clinical relevance appears to be less clear. The antibacterial effect of high-viscosity glass-ionomers frequently used with ART has been reported in *in-vitro*^10^^,^^16^ and *in-vivo*^27^ studies. The antibacterial effect on infected (decomposed) and affected (demineralised) dentine has been significantly increased when chlorhexidine was added to a high-viscosity glass-ionomer^27^. Such a finding is highlighted by Imazato^31^ (2009) as a positive innovation in restorative dentistry. This indicates that incorporation of 1% chlorhexidine diacetate into glass-ionomer used for ART is optimal for reduction of the level of bacteria in infected (decomposed) and affected (demineralised) dentine.

In-vitro studies have clearly shown that fluoride from glass-ionomers is released into enamel, dentine and the oral environment. Donly, et al.^17^ (1999) in an *in-situ* study demonstrated the remineralising effect of a glass-ionomer in artificially produced enamel carious lesions. The remineralising effect of high-viscosity glass-ionomer in dentine after 3 months has been evident in the increase of calcium, fluoride and strontium in affected dentine after cavity cleaning using ART^44^.

Several studies have demonstrated the antibacterial properties and remineralising effects derived from glass-ionomers used with ART. However, clinical trials are necessary to support the clinical relevance of such features that, applied to the ART concept, may help to control the onset or progression of carious lesions and to achieve a better integration of the restorative material into the cavity.

## CONVENTIONAL LOW-VISCOSITY VERSUS HIGH-VISCOSITY GLASS-IONOMERS

Many brands of (medium-) high-viscosity glass-ionomers have been developed and marketed for use with ART, although only a few of them have been tested in clinical trials. The ART meta-analysis^53^ concluded that the survival rates of ART restorations using high-viscosity glass-ionomers were superior to those using medium-viscosity glass-ionomers. Therefore, only high-viscosity glass-ionomers that have been field-tested in long-term follow up studies should be used with ART.

The flexural strength values reported in most studies that have compared different commercially available high-viscosity glassionomers was low. Such a finding, when extrapolated to a clinical situation, may be the reason for the relatively easy fracture of the material and the subsequent failure of the restoration^11^^,^^57^. Compressive strength, often used to measure the ability of the material to withstand masticatory forces, varied according to the brands of glass-ionomer tested, with the well-established high-viscosity glass-ionomer brands (Fuji IX, Ketac Molar, Ketac Molar Easymix) performing well^1^^,^^11^^,^^47^.

## HAND-MIXED VERSUS ENCAPSULATED GLASS-IONOMERS

Encapsulated high-viscosity glass-ionomer has been on the market for a decade or so. According to Dowling and Fleming^18^^,^^19^ (2008,2009), encapsulated anterior and posterior glass-ionomer restoratives outperform their hand-mixed equivalents with regard to the range of powder to liquid mixing ratios routinely encountered clinically. Therefore, if electricity is available, encapsulated high-viscosity glass-ionomers are preferable to hand-mixed glass-ionomers with ART. However, if electricity is not available, it is mandatory for the operator to use the correct liquid to powder ratio, in order to obtain optimal properties from the cement. Being careless and mixing less powder into the drop of liquid, as often happens in practice, will lead to a weak glass-ionomer and consequently, to a poor restoration or sealant.

The only study in which encapsulated high-viscosity glass-ionomer was used with ART showed a cumulative survival rate for single- and multiple-surface ART restorations in permanent teeth of 85% and 77% after 5 years^25^.

Dowling and Fleming^18^^,^^19^ (2008,2009) further conclude that anhydrous glass-ionomer restorative formulations are more susceptible to clinically-induced variability in hand-mixing, in contrast to conventional GI restorative formulations that contain a polyalkenoic acidic liquid. Therefore, if hand-mixed glass-ionomers are used for ART, using those with formulations containing the acid in the liquid is preferable to using those containing it in the powder. Thus, if encapsulated high-viscosity glass-ionomers can be used, these are to be preferred over hand-mixed high-viscosity glass-ionomers.

## RESIN-MODIFIED GLASS-IONOMERS

Incorporation of resin components into glass-ionomers results in better optical properties, control of the setting time by means of light curing, greater early physical strength and less susceptibility to dehydration. Compared to high-viscosity glass-ionomers, resin-modified glass-ionomers have higher values for flexural strength and diametric tensile strength^57^, and higher values for strength of tensile bonding to enamel and dentine^46^.

Resin-modified glass-ionomers would be suitable for use with the ART approach only when a light-curing device, whether with a cord or cordless, is available. A few clinical studies have investigated the success of resin-modified glass-ionomers with ART. Survival of single-surface ART restorations in primary teeth, using resin-modified glass-ionomers and placed by dental students, showed a success rate of 72% after 25-48 months^24^. The success rate of resin-modified glass-ionomers used for restoring single- and multiple ART-cleaned cavities in permanent teeth appears to be higher than for comparable high-viscosity glass-ionomers after one year^13^ and 2 years^21^.

The results of these few short-term studies are encouraging. Further research into the use of resin-modified glass-ionomers with ART is therefore warranted.

## NEWLY DEVELOPED RESTORATIVE MATERIALS

Physical properties of a newly launched fluorapatite containing glass-ionomer: glass-carbomer, were tested *in-vitro* in large class II ART restorations in permanent teeth. The material was compared with high-viscosity glass-ionomers and a resin composite. Class II ART cavities restored with glass-carbomer were not significantly more fracture resistant than comparable restorations using the conventional hand-mixed high-viscosity glass-ionomers, Fuji IX and Ketac Molar easymix. Further research is needed to assess the clinical potential of this new cement^34^.

Physical and mechanical properties in experimental modifications of a conventional medium-viscosity glass-ionomer were evaluated. Glass-ionomers containing N-vinylpyrrolidone (NVP), nano-hydroxyapatite and fluoroapatite were compared with the original glass-ionomer (Fuji II, GC). The results showed higher values for compressive strengths, diametral tensile strength and biaxial flexural strength and handling properties (working and setting time) for NVP-nanoceramic powder modified cements than for the control group^41^. Considering that this is a self-curing material with enhanced physical properties, this material, if marketed, could be an option for use with ART.
